# Malaria in the 21st Century: Global Disease Burden, Epidemiological Insights, and Strategic Control Approaches

**DOI:** 10.3390/biology15070575

**Published:** 2026-04-03

**Authors:** Basmah F. Alharbi, Mawahib A. Ahmed

**Affiliations:** 1 Department of Basic Health Sciences, College of Applied Medical Sciences, Qassim University, Buraydah 52571, Saudi Arabia; 2 Department of Medical Laboratories, College of Applied Medical Sciences, Qassim University, Buraydah 51452, Saudi Arabia

**Keywords:** malaria, disease burden, *P. falciparum*, rapid diagnostic tests, epidemiology

## Abstract

Malaria remains a major public health issue worldwide and a repeated cause of illness and death in tropical and subtropical areas. It is caused by protozoan parasites of the genus Plasmodium and transmitted through bites of infected female Anopheles mosquitoes, but it can also be transmitted via blood transfusions, organ transplants, and congenitally from mother to child. Despite decades of intervention efforts, millions of new cases and hundreds of thousands of deaths still occur each year, primarily in low- and middle-income countries. This review summarizes current epidemiological data on the global burden of malaria, mainly from the World Health Organization’s (WHO) World Malaria Report 2024 and Global Burden of Disease estimates. It brings together the latest evidence on worldwide malaria epidemiology, regional trends, determinants, and control strategies, with a particular focus on socio-economic factors, intervention methods, and emerging challenges such as drug resistance, climate change, and limited funding. Disease prevention and management require global, multifactorial approaches that are tailored to the local environment. Strengthening health education with locally relevant knowledge is important to improving outcomes in primary health prevention, secondary health prevention, and tertiary health prevention. The review concludes with a discussion of policy priorities needed in the future to meet the WHO Global Technical Strategy goals for malaria elimination by 2030.

## Simple Summary

Malaria is a significant health issue in the world, and it results in numerous diseases and fatalities, particularly in developing economies. The following review summarizes the latest epidemiological reports from WHO and GBD, including patterns and regions of malaria distribution, the impact of social and economic determinants, and key control measures. It also brings up issues like resistance to drugs, climate change, and budget deficits and emphasizes the necessity of prevention and health education with a particular emphasis on context and level. Policy recommendations have been given at the end of the review to assist in meeting the WHO malaria elimination targets by 2030.

## 1. Introduction

Malaria is one of the oldest and most deadly infectious diseases known to man, having been caused by protozoan parasites of the genus *Plasmodium* and transmitted by the bite of infected female Anopheles mosquitoes [1]. Among the five Plasmodium species that are pathogenic to humans (*P. falciparum*, *P. vivax*, *P. malariae*, *P. ovale,* and *P. knowlesi*), *P. falciparum* is the most deadly, with most severe cases and deaths being recorded, especially throughout sub-Saharan Africa [2]. *Plasmodium vivax* is predominant in South and Southeast Asia and in some localities of Latin America. Its dormant hepatic stage (hypnozoite) causes relapse infections, thus complicating the elimination of malaria [3].

**Figure 1:**
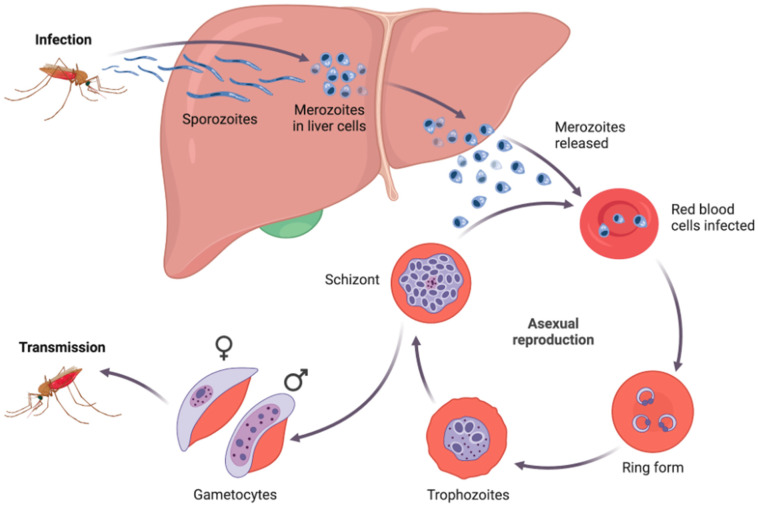
Malaria transmission cycle and infection depiction, liver stage development, erythrocyte invasion, and further transmission stages of Plasmodium parasites. Created with BioRender.com and adapted from [16].

Only Anopheles mosquito bites are capable of spreading malaria. There are 512 species of Anopheles known to exist in the entire world, of which 50 have only been provisionally identified and need further description. Sixty-eight species of Anopheles can spread the *Plasmodium* parasite to humans [4]. Every species of Anopheles mosquito has a preferred method of reproduction, and they all reproduce in water. The life cycle of *Plasmodium* is behind a high degree of evolutionary adaptation, where there is a shift between asexual propagation in the human host and sexual reproduction in the mosquito vector [5]. Upon entering the bloodstream through a mosquito bite, sporozoites quickly enter the hepatocytes, where they cause a clinically silent pre-erythrocytic phase as depicted in Figure 1. After release of merozoites, the erythrocytic stage develops with repetitive pyrexial paroxysms with chills and anemia, in which the parasite invades and lyses erythrocytes [6]. Adherence of *P. falciparum*-infected erythrocytes to vascular endothelium initiates severe complications associated with *P. falciparum* infection, such as cerebral malaria, severe anemia, and multi-organ dysfunction. Gametocytes (sexual stages) are taken up by a feeding mosquito, where fertilization, ookinete formation, oocyst formation, and migration of sporozoites towards the salivary glands are completed, and the cycle is completed in the onward transmission [7]. In areas where anthropophilic mosquitoes are prevalent and mosquito lifespans are longer, the parasite has more time to finish developing inside the insect. The parasites’ life cycle is a complicated process that takes place in both mosquito vectors and vertebrate hosts [8].

The disease is a prime example of the interplay between the biological, ecological, and socioeconomic factors. Malaria develops in areas where poverty, poor health infrastructure, and climatic conditions meet. Transmission intensity is a function of climatic variables, including temperature, precipitation, and humidity, which affect mosquito breeding and parasite development [9]. Despite significant advances in the past 15 years, mostly through vector control and artemisinin combination therapy (ACTs), the global reduction in malaria morbidity has stagnated in recent years. Emerging drug resistance and structural weaknesses of health systems, and the impacts of climate vagaries and conflict, threaten to reverse previous achievements [10]. A similar analysis, the Global Burden of Disease (GBD) 2023 analysis, also reported malaria as one of the leading causes of infectious disease morbidity and mortality in low-income countries [11].

In East Africa, malaria is still a major health issue that gets exacerbated by several environmental, financial, and infrastructure issues. Favorable mosquito breeding circumstances, widespread poverty, considerable population mobility, and a lack of healthcare resources are important contributing factors [12]. Malaria transmission varies due to the region’s natural diversity, which includes mountains, river basins, including coastal zones. This underscores the necessity for locally specific therapies [13].

Between 2000 and 2015, major progress was made with global malaria cases reduced by 37% and deaths by 60% as a result of the increase in vector control (insecticide impregnated nets and indoor residual spraying), widespread adoption of ACTs, and improved diagnostics. However, this momentum has been lost since 2015 [14]. The most recent World Malaria Report states that there were 241 million reported cases of malaria in 2020 and 227 million cases in 2019. With an emphasis on enhancing basic healthcare regionally, early disease detection, prompt treatment, and disease prevention, the WHO is in charge of a global malaria control program [15]. The prevalence of malaria seems to be declining compared to around ten years ago. The prevalence, epidemiology, and regional trends of malaria are all discussed in the current review article.

## 2. Global Burden of Malaria

Estimating the prevalence of malaria is difficult, especially in low-income countries with poor data collection and reporting. The global malaria prevalence may be affected by incomplete and inconsistent reports from individual healthcare facilities. In countries where malaria is highly common, cases are often underdiagnosed, and mild symptoms of chronic malaria can lead to misdiagnosis. Conversely, over diagnosis is also possible [17]. In reality, not every reported case of malaria is confirmed by microscopy or other tests, such as rapid diagnostic tests (RDTs). Additionally, febrile illnesses caused by other factors may be mistaken for malaria in highly endemic regions.

Ultimately, all malaria cases should be confirmed using microscopy as the preferred and accessible reference method for local populations, rather than relying solely on rapid diagnostic tests, which may have limitations in sensitivity and interpretation, particularly in hyperendemic regions [18]. Moreover, positive cases should be monitored for at least 21 days following treatment [19].

### 2.1. Incidence and Prevalence

The number of malaria cases per 1000 people at risk of the disease (referred to as the global malaria incidence rate) was estimated at nearly 58 cases per 1000 population in 2022. This is a moderate decline from figures for 2000 (approximately 81 per 1000), but progress has been flat since 2015, suggesting the persistence of foci of transmission and emerging problems due to vector resistance to insecticides and parasite resistance to artemisinin-based treatments [11].

The prevalence of malaria is high in Sub-Saharan Africa bore the brunt of all malaria cases globally, at about 94% of cases, due to favorable environmental factors for breeding of the mosquito, the prevalence of *P. falciparum*, and socio-economic-to-hindering efforts of effective malaria prevention. *P. vivax* continues to be the predominant species outside Africa in Southeast Asia and sections of Latin America [20]. Even though case levels in Asia and the Americas have decreased over the last 2 decades, the results have been uneven and fragile.

### 2.2. Mortality Patterns

More than three-quarters of children who die from malaria are under 5 years old, indicating immature immunity and limited access to timely diagnosis and treatment. Similarly, pregnant women are also at greater risk because physiological and immunological changes allow the parasite to sequester in the placenta. Mortality is highest in areas with high transmission, low health coverage, and ongoing war [21]. The regional malaria trend as per the WHO report in 2023 is provided in Table 1.

### 2.3. Disability Adjusted Life Years (DALYs)

Malaria accounts for an estimated 45 million DALYs each year, including premature deaths and years lived with disability. The disease is among the leading causes of DALYs in sub-Saharan Africa, alongside HIV/AIDS and tuberculosis. In addition to clinical malaria, the burden of health is increased by sequelae of chronic malaria, such as chronic anemia, cognitive impairment in children, and maternal morbidity [22].

Using the mapping of the above data from the WHO and GBD, there were dense concentrations of transmission found in Central and West Africa, the Amazon Basin, Papua New Guinea, and coastal Southeast Asia. Regions that made significant gains in the past, like India and Southern Africa, are now experiencing localized resurgence associated with resistance, climate change, and funding shortfalls [23]. WHO malaria report of 2024 [24] and that by Hyun-II Shin et al., 2024 [25] is given in Table 2.

### 2.4. Tendencies and Issues of the Present Day

**Figure 2:**
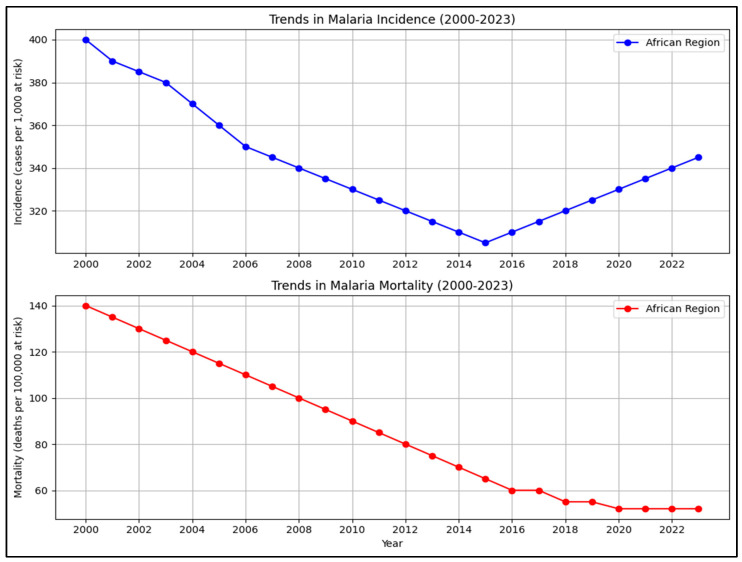
Presents the trends in malaria incidence and mortality in the WHO African Region from 2000 to 2022 as per the data adapted from WHO World Malaria Report 2023/2024.

**Figure 3:**
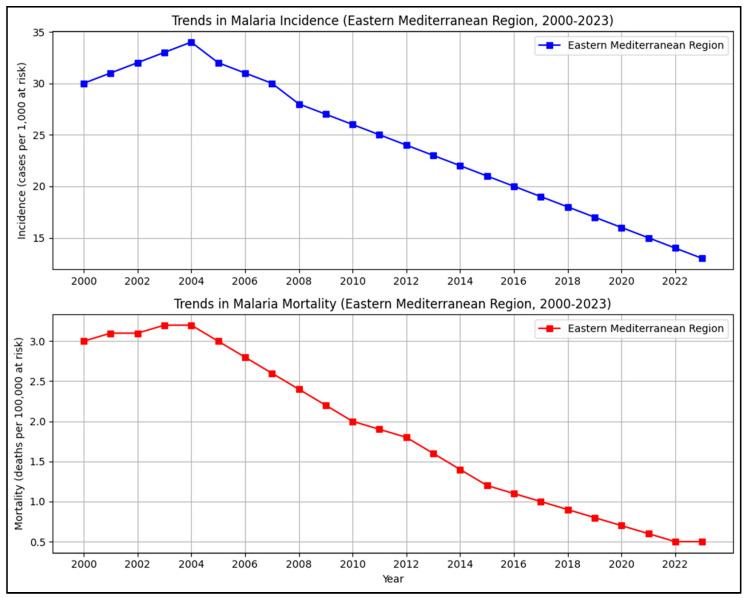
Presents the trends in malaria incidence and mortality in the WHO Eastern Mediterranean Region from 2000 to 2022 as per the data adapted from WHO World Malaria Report 2023/2024.

**Figure 4:**
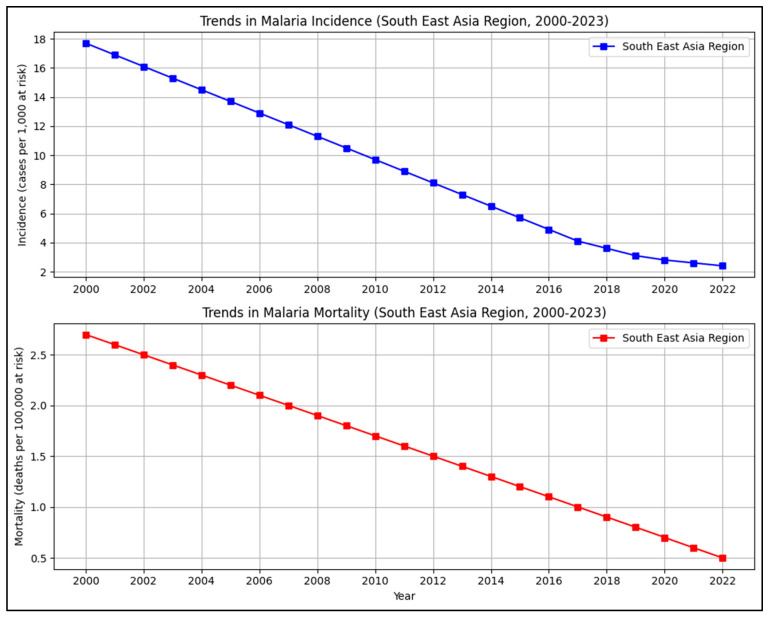
Presents the trends in malaria incidence and mortality in the WHO Southeast Asia Region from 2000 to 2022 as per the data adapted from WHO World Malaria Report 2023/2024.

While the malaria control program has prevented millions of deaths since 2000, advancement towards elimination has been hindered in recent years. The factors contributing to the gradual decline include climate change, which is increasing vector habitats; rapid urbanization without proper vector management; insecticide resistance in Anopheles species; and drug resistance in *P. falciparum* and *P. vivax*. The current malaria statistics illustrating these trends can be seen in Figure 2, Figure 3 and Figure 4 [25]. Such problems risk undoing gains made in the early 2000s under a global strategy of rolling back malaria (RBM Partnership) and the Global Fund [26,27].

## 3. Regional Trends

Malaria is still an important global public health problem, in which the elimination pace has slowed in recent years [28].

### 3.1. Sub-Saharan Africa

Africa remains the epicenter of malaria burden, with over 90% of global malaria cases and deaths occurring in the WHO African Region. Four high-burden-to-high-impact countries, Nigeria (27%), Democratic Republic of the Congo (12%), Tanzania (6%), and Niger (5%), accounted for more than 50% of the mortality, and Nigeria, Democratic Republic of the Congo, Uganda, Mozambique, and Niger combined accounted for 55% of the burden in the region [29]. While malaria mortality rates declined substantially between 2000 and 2015, malaria deaths remain significant in Africa, and mortality has not reached zero after 2020 [30]. Cabo Verde was certified malaria-free by the WHO on 12 January 2024, becoming one of the countries officially recognized as having eliminated indigenous malaria transmission [30,31]. Pyrethroid resistance in Anopheles gambiae is close to fixation in Western Africa, and kdr west (L1014F) and kdr east (L1014S) alleles are found at more than 90% frequency in Nigeria and Kenya, respectively. Metabolic resistance via overexpression of CYP6P9a/R subsequently undermines piperonyl butoxide nets and reduces mortality by 40%, as observed in resistant hotspots [31]. Artemisinin based combination therapy efficacy remains above 95% in most settings; however, parasite clearance failure (day 3 positivity > 10%) with a Kelch13 C580Y mediated partial resistance has been reported in Rwanda, Uganda, and Northern Tanzania [32]. There are a few places (e.g., eastern Democratic Republic of the Congo, Sahel), where the coverage of long lasting insecticidal nets is less than 30% and seasonal malaria chemoprevention is less than 50%, and increased vulnerability [33]. Genomic surveillance exposes a *P. falciparum* hrp2/3 deletion in 15% of Eritrean isolates that puts rapid diagnostic test sensitivity at risk. Dual active ingredient nets (chlorfenapyr pyrethroid) have shown an entomological impact of 40–50% higher in the resistant areas, and spatial repellents (transfluthrin) were trialed in Northern Nigeria; however, scale up is limited by cost (USD 5–7 per net) and fragility of supply chains [34]. Research priorities: (1) CRISPR-based gene drive trials, (2) monoclonal antibody prophylaxis (L9LS) on the condition of pregnancy, and (3) climate resilient forecasting that merges the El Niño driven transmission spikes [35]. In Africa, in particular, it is important to focus more on accurate diagnosis and management of malaria in children under 5 years of age because their health carries important social and economic ramifications for the future workforce and development of the country [36].

### 3.2. Southeast Asia

Optimistic results have been seen in India, Myanmar, and Indonesia as a result of well-planned national elimination strategies. “Zero indigenous cases of malaria in India by 2030”: Mission to Control and Eliminate Malaria in India, also known as the “National Framework for Malaria Elimination 2016–2030” [37]. Nevertheless, declining infections and transmission of *P. vivax* in borderline areas are still significant problems. Greater Mekong Subregion (GMS) exemplifies both success and challenge. Some countries, such as Thailand and Vietnam, are on the verge of elimination [38]. Still, others have ongoing *P. falciparum* resistance, which threatens the efficacy of current therapy for the whole world. This area had some 4 million cases and 6000 deaths in 2023, a great success story compared to other areas of the world. Malaria prevalence and incidence decreased by 87% and 88.8%, respectively (from 17.7 to 2.3 incidence per 100,000 recorded cases; from 2.7 to 0.3 deaths per year per 100,000, and per 100,000 live births) [39]. Reductions have been maintained through national elimination campaigns, and ACT rollout in countries such as India, Bangladesh, Indonesia, and Nepal [40].

### 3.3. South America

The Amazon Basin contributes about 90% of the 0.9 million cases of malaria reported in the Americas, with 58%, 22%, and 12% of cases reported from Brazil, Venezuela, and Colombia, respectively [41]. This moved back by 25% between 2020 and 2023, reversing a 50% decrease that was seen between 2000 and 2015 [42]. Mosquito habitats have been enlarged by political instability, mining, migration, and deforestation, and control has been impeded [43]. The economic crisis and population displacement in Venezuela have reversed past gains and led to transboundary transmission to neighboring countries. *P. vivax* is prevalent and represents 75% of infections, and the drug was also found to be resistant to chloroquine in 40% of infections mediated by Y976F, which is a common mutation in the gene pvmdr1 [44]. In Venezuela in 2022 distribution of insecticide-treated nets (ITNs) in mining camps was less than 30%, and stockouts of artemisinin combination therapies were greater than 60% [45].

### 3.4. Eastern Mediterranean

Based on the WHO 2023 Malaria report [24], the infection has not been eliminated in the Eastern Mediterranean, and about 4.5 million people in the region continue to become infected with the parasite, with Sudan contributing 60%, Yemen 25%, and Pakistan 10%. Armed conflict seriously interferes with intervention delivery. In Sudan, the conflict from 2023 to 2025 displaced an estimated seven million people and led to the collapse of more than 70% of health facilities in Khartoum and Darfur, which in turn caused a 40% increase in mortality attributed to malaria [46]. The blockade of Yemen limits the importation of insecticides, which is leading to pyrethroid resistance among Anopheles arabiensis (85% prevalence) [47]. Pakistan’s 2022 malaria outbreak was exacerbated by floods, which reached 0.5 million cases and showed shortages of RDTs; 80% of the cases were caused by *P. vivax*; and a lack of primaquine adherence below 50 percent, due to a lack of G6PD screening [48]. Some of the research priority areas in this region include: (1) the development of conflict adaptive micro planning methodologies; (2) deployment of thermostable diagnostic tools; and (3) the integration of malaria interventions into polio and measles platforms, which are already in place [49].

### 3.5. Western Pacific Region

The western Pacific region was reported with 1.7 million cases with 3400 deaths in the year 2023 due to the deadly infection of malaria [50]. Based on the WHO 2023 report, in recent years, after earlier declines, incidence and mortality have increased by 35% and 33%, respectively, since 2015, largely due to increases in Papua New Guinea and the Solomon Islands. The WHO Global Technical Strategy 2030 target has still not been met in this region.

### 3.6. European Region

**Figure 5:**
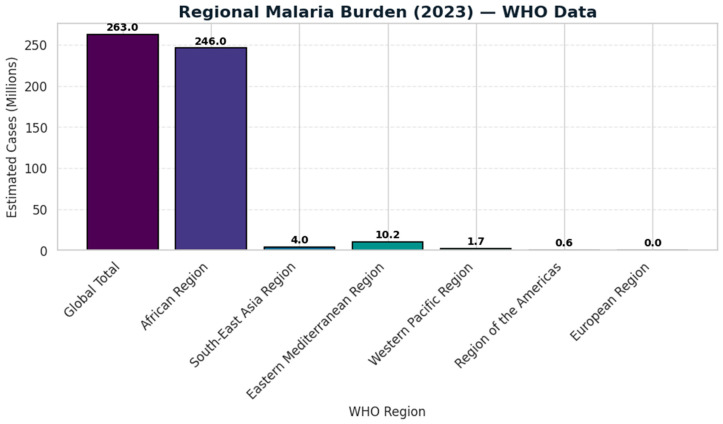
Exhibit the malaria cases based on the WHO region, WHO World Malaria Report 2023/2024.

The European Region has been malaria-free since 2015 and reported no cases or deaths in 2023. It is estimated that 5000 imported cases per year are treated by the use of point-of-care diagnostic tests among travelers [51]. The WHO regional malaria burden in 2023 is presented in Figure 5.

## 4. Determining the Burden of Malaria

### 4.1. Drug and Insecticide Resistance

The resistance of malaria parasites to antimalarial drugs is the most immediate biological threat to malaria control. National treatment policies should be set on the basis of the local resistance profiles of malaria parasites to be effective in the management and maintenance of drug-sensitive populations. For example, chloroquine was phased out in some areas as a result of widespread resistance, but, after a few years of halted administration of this drug, the sensitive strains of the parasite reemerged because of the lack of selective drug pressure. The implementation of such adaptive policies demands pre-existing knowledge on profiles of resistance, and continued funding for ongoing epidemiological surveillance [52]. Artemisinin partial resistance (ART R), which was first described in the Greater Mekong Subregion in 2008, has by now firmly established itself in Cambodia, Vietnam, Thailand, and Myanmar [53]. Pfkelch13 mutations, of which C580Y is a good example, cause a delay in parasite clearance of over five hours in 20–60% of clinical episodes. Failure of partner drugs such as piperaquine has resulted in the emergence of multidrug-resistant lineages, thus reducing the efficacy of ACT from >95 to <70 per cent in selected foci [54]. In Africa, ART R emerged independently in Rwanda (2018), Uganda (2020), and Eritrea (2022) with the validated markers: kelch13 R622I. Although failure of clinical response is rare (less than 5%), delayed parasite clearance that occurs after treatment with ARTR prolongs gametocytemia and increases transmissibility [55]. Resistance to sulfadoxine pyrimethamine (SP) has undermined intermittent preventive treatment in pregnancy (IPTp) in East Africa, where quintuple and sextuple dhfr/dhps mutants dominate [56].

The resistance phenomenon creates a vicious circle where high infectious periods promote a high level of transmission, which, in turn, justifies an increase in intervention coverage, accelerating selective pressure [57]. Triple ACT regimens (artemisinin plus two elimination partners that slowly disappear) are now being evaluated in phase III trials but are lagging, and the higher cost (about USD 2–3 per dose as compared with USD 1 for standard ACTs) is of significant equity concern [58]. Genomic surveillance, which has been supported by the Plasmodium Resistance Network, is a backbone of malaria control, but only 15 high-burden countries have functional genomic surveillance infrastructures in place [59].

### 4.2. Poverty

Poverty influences the risk of malaria through the dimensions of exposure, susceptibility, and access barriers. In sub-Saharan Africa, 405 million people living below USD 2.15 per day carry 90% of the world’s malaria cases [60]. Overcrowded residences without screening, proximity to breeding sites (periodic peri-urban rice fields), and occupational exposure, in particular nighttime agricultural work, increase the biting rate [61]. In Nigeria, the odds ratio for having a parasitemia is 2.5 times higher in the lowest quintile of the wealth distribution. Malnutrition, especially stunting, which afflicts 33% of the African children, increases the risk of severe malaria by two-to-four times due to the loss of immunity and tolerance to anemia [62]. Poverty also reduces the uptake of preventative interventions: only 45% of eligible children are reported to be using ITNs in Africa, and the poorest quintile is half as likely to have access to one [63]. In pregnant women, sequestration of parasites in the placenta is a cause of low birth weight and anaemia in mothers, while intermittent preventive treatment in pregnancy (IPTp) coverage remains stagnant at 35% in settings of moderate to high transmission [64]. Out-of-pocket expenditures for diagnostics (rapid test USD 0.50–1) and treatment are a disincentive to seeking care; catastrophic expenditure (more than 40 percent of non-food expenditure) affects 60% of malaria-affected households in rural Ghana [34]. Cash transfers and voucher interventions, such as the malaria voucher program in Tanzania, have shown further evidence for the feasibility of poverty alleviation as a complementary intervention to address ITN ownership by 20–30% [65].

### 4.3. Health Inequities

Malaria disproportionately affects children under 5 years of age, pregnant women, and marginalized people [66]. For instance, 76% of deaths attributed to malaria occur in children under 5 years of age, as immature immune systems and delays in care are the main risk factors [67]. Equity issues in access to treatment: In patriarchal societies, women can often be restricted in their mobility, and thus delay medical care, which leads to 1.8-fold increased odds for severe malaria [68]. Ethnic minorities and migrants are victims of exclusionary practices [69]. In Myanmar, IDP Rohingya communities have demonstrated cases of malaria five to ten times higher than the national average of incidence due to both crowded camps and limited access to healthcare [70]. In Brazil, indigenous Yanomami populations are affected by hyperendemic transmission from gold mining (illicit mining, in this case), with transmission rates of more than 300 per 1000 persons [71]. HIV doubles the risk of malaria and triples the risk of serious malaria; currently, only 60% of co-infections in pregnant women are treated with IPTp [72]. Genetic differences include the fact that, in addition to defending against approximately 90% of severe *P. falciparum* cases, individuals with the sickle cell trait have a probability of hemolysis if given primaquine, whereas glucose 6 phosphate dehydrogenase deficiency increases the risk of hemolysis [73].

### 4.4. Climate and Environmental Change

Climate modulates the Anopheles vectorial capacity through temperature, rainfall, and humidity [74]. Optimal *P. falciparum* development temperature is 25–30 degrees, with one degree centigrade increase in temperature prolonging the seasons of transmission by one to three months in highland fringe regions such as Kenya and Ethiopia [75]. The floods of 2019–2020 in the Horn of Africa led to the introduction of Anopheles [76]. Stephensi (an urban adapted vector) that has now settled in Djibouti, Sudan, and Nigeria, threatening malaria-free urban areas [77]. Deforestation and irrigation provide new breeding sites. Road construction in the Amazon has been linked to a 10 percent–20 percent incidence [78]. Sea-level rise in the Pacific is pushing people into malaria-receptive areas, as observed in Papua New Guinea [79]. There is a lack of funding to support larval source management and next-generation ITNs, for example, which have a clear role to play in promoting climate-resilient tools.

### 4.5. Conflict and Humanitarian Emergencies

**Figure 6:**
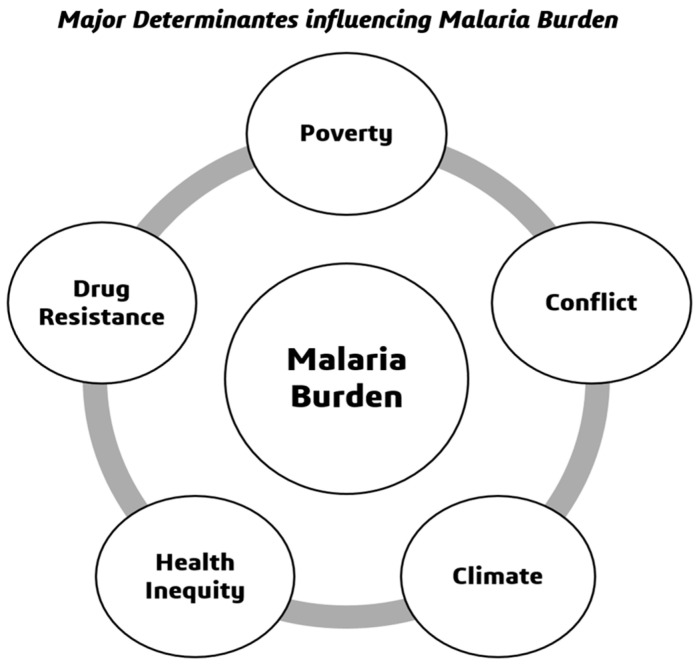
Depicts the major determinants increasing malaria burden globally.

War has a disruptive effect on health systems, vector control, and the immunity of populations. In 2023, 70% of malaria deaths were confined to 12 countries affected by the crisis [80]. Yemen’s civil war has had the effect of reducing ITN distribution by 60%, with case numbers increasing 300% since 2015 [81]. In the Democratic Republic of Congo, 25 million ITNs expired undistributed amidst the violence of militias [82]. Mass displacement into camps, such as 1.2 million residents in Cox’s Bazar in Bangladesh, creates ideal transmission conditions: stagnant water bodies, no IRS, and low ITN coverage [83]. Cross-border movements contribute to the spread of resistant parasites; ART R prevalence in Myanmar endangers elimination goals in India [84]. Lack of access for humanitarian actors hindered outbreak responses; in the conflict in Tigray (2020–2022), malaria cases rose by 400% after health facilities were shut down [85]. Malaria persistence to this day is a reflection of a syndetic of drug resistance, poverty, inequity, climate change, and conflict [86]. Combined interventions, such as genomic surveillance, poor financing mechanisms, gender responsive programming, and climate smart vector control tools, and enhanced humanitarian public health partnerships are needed to restore progress towards the 2030 elimination goals [87]. Figure 6 depicts the major determinants of increasing malaria burden globally.

## 5. High-Risk Populations

Malaria causes a disproportionate burden in unique subpopulations with physiological, immunological, or socio-environmental vulnerabilities that increase both the incidence and case fatality ratio CFR [88]. This section has four key groups of affected people under the age of 5, pregnant women, forcibly displaced people, and immunocompromised people, and discusses the pathophysiology, clinical symptoms, and evidence-based interventions in a format that is suitable for use with both academic and programmatic audiences.

### 5.1. Children Under Five

Young children under the age of 5 are the most vulnerable group in malaria-endemic areas. According to the WHO, almost three-fourths of all malaria fatalities take place in this age group [67]. Their immune systems are not fully developed, and the protection they receive is broad but temporary, usually lasting only a few months, from maternal antibodies transferred before birth [89]. Once the parasites are injected into a person by an infected mosquito bite, the parasites multiply quickly in the bloodstream, and the body cannot combat the high parasitemia [6]. This frequently results in severe malaria, which presents as anemia, hypoglycaemia, metabolic acidosis, or cerebral complications, which can be fatal in the absence of prompt treatment [90]. Chronic malaria-associated anemia further limits oxygenation to important organs, such as the brain, driving neurocognitive delays, growth retardation, and learning impairment [91]. Children who survive severe episodes are thus vulnerable to long-term health and cognitive consequences [92]. From the public health point of view, among these interventions, the Intermittent Preventive Treatment in Infants (IPTi), using sulphadoxine pyrimethamine and associated with routine immunizations, has proved effective in significantly reducing the burden of clinical malaria and anemia [93]. Similarly, Seasonal Malaria Chemoprevention (SMC), which is being implemented to a great extent in the Sahel region of West Africa, provides antimalarial drugs at regular intervals during peak transmission seasons [94]. These strategies, combined with the use of Insecticide Treated Nets (ITNs), early diagnosis, and timely treatment, have greatly reduced malaria deaths in children. However, gaps in coverage in several endemic areas, the emergence of resistance to existing drugs, and weak health systems are still obstacles, especially in rural and conflict-affected areas [95].

### 5.2. Pregnant Women

Pregnancy causes massive immunological changes to provide a fertile ground for sequestration of *P. falciparum* in the intervillous space of the placenta [96]. Parasite adhesion to chondroitin sulfate A through VAR2CSA-expressing infected erythrocytes leads to impaired nutrient and oxygen transfer, the precipitation of maternal anaemia, fetal growth restriction, premature delivery, and perinatal loss [97]. Primigravidae are most burdened by the absence of variant-specific immunity. Intermittent preventive treatment in pregnancy (IPTp) of sulfadoxine pyrimethamine, started in the second trimester and repeated monthly, has a significant effect on placental parasitaemia and adverse birth outcomes [98]. Co-administration of ITNs has the added advantage of dual suppression (vector and parasite) [99]. The community-based delivery model overcomes the facility-based bottlenecks and helps to provide superior coverage in rural and peri-urban settings [100].

### 5.3. The Problem of Displaced Populations and Refugees

Another particularly vulnerable group of people is refugees, internally displaced persons (IDPs), and migrants [101]. Huge populations forced to move by armed conflict, natural disasters, and political collapse will migrate to overcrowded shelters, poor sanitation conditions, and limited access to health care, which is conducive to malaria transmission and outbreaks [102]. In many cases, displaced populations migrate from low- to high- but still endemic areas where they have no acquired immunity and are thus at higher risk of severe disease [103]. In addition, disruptions in national malaria control programs, such as failures in vector control, drug stockouts, and disrupted surveillance systems, contribute to the problem [104]. A significant number of refugee camps and temporary settings do not have sufficient insecticide treated nets, indoor spraying, and diagnostic facilities, and as such are left vulnerable [105].

### 5.4. Immunocompromised Individuals

Individuals who suffer from HIV/AIDS and other immunosuppressive conditions are at risk of developing severe malaria. Greater parasite burdens and the increased likelihood of failure to cure are due to the interaction of biological and environmental stresses and malaria HIV co-infection, making integrated malaria HIV management services in endemic regions more critical [106]. Other forms of immunosuppression, such as malnutrition, anticancer therapy, or autoimmune disorders, also increase susceptibility to conditions other than HIV [107]. For example, children are more likely to have severe malaria if they are malnourished, as this increases their susceptibility to the disease by affecting the immune system and the quality of red blood cells [108].

## 6. Social Determinants of Health and Socioeconomic Impact

The burden of malaria goes far beyond health measures, with serious social and economic costs to individuals, families, and nations [109]. Direct medical costs include spending on diagnostics, drugs, and hospital care. Indirect costs occur due to productivity loss, absenteeism from schools, and reduced agricultural yield. In countries with high burdens, malaria can slow annual growth 1.3 by percentage points of GDP growth per year, a figure that adds to the problem of poverty and hinders development [110].

The disease also interferes with education, as children are absent from school either because they are ill or tending to other members of the family [111]. Recurrent episodes lead to poor cognitive development and learning outcomes. In pregnant women, malaria causes maternal deaths and poor infant health, thus ensuring the perpetuation of inter-generational disadvantage [66]. Tourism and foreign investment tend to decrease in areas that are seen as malaria-endemic, therefore limiting economic opportunities. Consequently, malaria control is not only a matter of health necessity, but also the driving force of social and economic transformation [112].

## 7. Interventions and Progress

Malaria control is at a new stage where the traditional vector control, innovative vaccines, and reinforced surveillance systems work synergistically to reduce transmission. Despite some notable progress across the world, however, malaria remains a significant problem in areas with sparse health infrastructure, increasing insecticide resistance, and sociopolitical instability [113]. The following section summarizes major intervention strategies and progress throughout the world.

### 7.1. Vector Control

#### Vector Control via ITNs and via Indoor Residual Spray (IRS)

**Figure 7:**
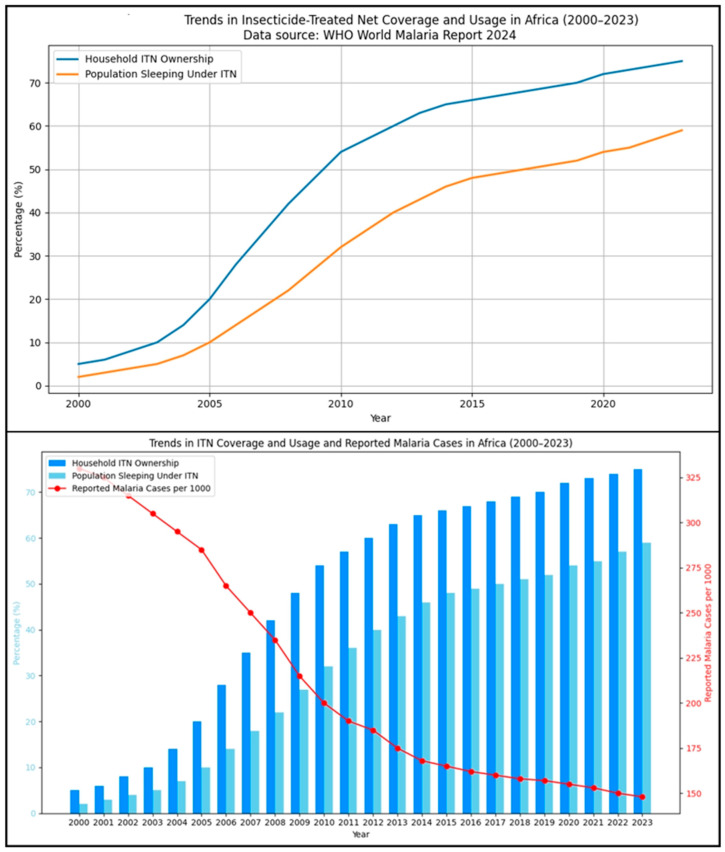
Illustrates the population using the ITNs and the curve of reported malaria cases from 2000 to 2023, according to the recent WHO malaria report.

ITNs are the mainstay of preventing malaria infection from mosquito bites because they offer both a physical and chemical barrier against mosquitoes [114]. According to WHO (2023), ITN ownership across sub-Saharan Africa has now surpassed 60 percent, and millions of infections and countless lives have been saved [115]. ITNs are particularly effective in children under 5 and pregnant women, who are the most vulnerable people. But with the widespread pyrethroid resistance in Anopheles mosquitoes, these gains could be lost [116]. In response, next-generation nets treated with piperonyl butoxide (PBO) or incorporating two insecticides, chlorfenapyr and alpha-cypermethrin, have been introduced [117]. These innovations have proved the ability to bring back protection to resistant areas, as has been proven by large-scale field trials in Tanzania and Burkina Faso [118]. ITN and IRS coverage, as well as larval source management, are highlighted for the WHO African Region because this region bears the overwhelming majority of global malaria cases. In high-burden African countries, these interventions remain the primary, widely implemented, and effective vector-control strategies [119]. IRS is complementary to ITNs in that it kills mosquitoes that rest indoors after feeding. This community-wide protection will save tremendous amounts of vector density and longevity [120]. Countries like South Africa, Mozambique, and Zanzibar have used IRS to get to the point of interrupting transmission and bordering on elimination [121]. The major obstacles are cost, logistical, and resistance to conventional insecticides like DDT, carbamates, and organophosphates [122]. Modern IRS programs are moving towards using novel insecticides such as clothianidin and pirimiphos methyl, under rotational schemes to avoid buildup of resistance to these insecticides [123]. Integrating IRS with ITN through Integrated Vector Management or IVM maximizes impact and also promotes environmental safety. Figure 7 shows the population using the ITNs and the curve of reported malaria cases from 2000 to 2023. Table 3 shows vector control interventions, limitations, and recent advancements of each intervention.

### 7.2. Case Management

Speedy and efficient diagnosis will help to avoid progression of the disease and to reduce transmission [124]. The advent of Rapid Diagnostic Tests or RDTs has brought about a revolution in the management of malaria, particularly in rural and resource-limited settings [125]. These tests are available in less than 20 min to give the result, allowing treatment at the community level without access to a laboratory [126]. They also reduce the unnecessary use of antimalarial drugs and help to contain drug resistance. Within the health facilities, microscopy is currently the diagnostic gold standard, providing quantification of parasites as well as species identification [104].

### 7.3. Effective Treatment

ACTs are the first-line choice for the treatment of Plasmodium falciparum infection around the world. They combine a fast-acting form of artemisinin with a slower partner drug to provide prevention of resistance. ACTs have undergone spectacular success, although artemisinin-resistant strains in Southeast Asia and East Africa pose an increasing challenge [127]. For *P. vivax*, the treatment of eradication consists of the elimination of the latent stage of hypnozoites in the liver with primaquine or tafenoquine. These schedules are effective in preventing relapse, but hemolytic complications are avoided by G6PD screening [128].

### 7.4. Vaccines

#### 7.4.1. Vaccines RTS, S/AS01 (Mosquirix)

The RTS, S/AS01 vaccine was approved by WHO in 2021, and it is a landmark in preventing malaria [129]. Pilot programs in Ghana, Kenya, and Malawi showed a 30–40 percent drop in severe malaria among the vaccinated children [130].

#### 7.4.2. R21/Matrix M

A new vaccine, called R21/Matrix M, developed by the University of Oxford and the Serum Institute of India, performed similarly in efficacy in Phase III trials (around 75 percent efficacy) [19]. It is cost-effective, easier to produce, and is already being rolled out in countries such as Nigeria, Ghana, and Burkina Faso [131]. While neither RTS, S nor R21 alone can eliminate malaria, both are potentially useful complementary tools which, when combined with ITNs and ACTs, can reduce the malaria burden substantially [132].

### 7.5. Elimination Successes and International Initiatives

Several countries have become certified by the World Health Organization (WHO) as malaria-free, such as China in 2021, El Salvador in 2021, and Argentina in 2022. These successful communities and areas show that elimination is possible with strong political will, strong surveillance, and community action. In fact, the WHO’s “High Burden to High Impact”, also termed as an HBHI project, focuses on just 11 African nations, which account for 70% of the global burden of all countries, and which prioritizes evidence-based, locally adapted interventions as the best approach [133].

## 8. Identifying Issues and Changing Trends

More than 2 decades since the WHO started moving towards a malaria elimination world, the world trajectory has slowed markedly since 2015. Many endemic areas are still experiencing ongoing and emerging conflict situations that negatively affect prevention, treatment, and surveillance [134]. The following key challenges reflect the key drivers constraining achievement of the malaria-free targets as set out in the WHO Global Technical Strategy for Malaria (2016–2030).

### 8.1. Resistance Issues

The outbreak and spread of parasite resistance and vector resistance are the most pressing biological threats [135]. Artemisinin resistance, which was first reported in the Greater Mekong Subregion, has been confirmed in areas of East Africa (Eritrea, Uganda, and Rwanda) and poses a risk to the effectiveness of ACTs, which form the key to the treatment of malaria [136]. In addition, resistance to partner drugs (e.g., piperaquine, lumefantrine) has made therapy further complicated, and thus there is an urgent need for triple ACTs (TACTs) and novel antimalarial scaffolds (WHO, 2024). There has been a widespread spread of insecticide resistance in Anopheles gambiae and Anopheles funestus to pyrethroids, mainly the class used in ITNs [137]. Based on the WHO 2023 data, the sub-Saharan region was detected with more than 70% drug resistance. The lack of control is due to the combined impact of drug resistance and insecticide resistance, which puts the success of treatment and vector control in jeopardy, as a threat to undo years of progress.

### 8.2. Poor Health Systems

Many high-burden countries have fragile health systems that are characterized by low surveillance and diagnostic coverage, and, hence, low reporting. Supply chain issues resulted in a regular lack of stocks of ACTs and RDTs [138]. There was a paucity of trained personnel, particularly in remote and conflict-affected areas, as well as a lack of strong linkages of malaria services with primary healthcare systems. Systemic fragilities in vector control were further revealed; efforts were redirected and concentrated, and disturbances occurred in vector control and data capture during COVID 19 [139]. Thus, consequently, malaria transmission in most endemic countries is susceptible to a “rebound” effect after temporary service breakdowns.

### 8.3. Climate Change

Malaria epidemiology is changing all over the world in response to climate variability and environmental change [13]. A warmer world, more precipitation, and altered relative humidity have the potential to expand the ecological niche of Anopheles mosquitoes and make previously malaria-free areas highland and temperate susceptible to malaria [140]. As per the IPCC AR6 report and WHO (2024), there is strong evidence that extreme weather-related events (i.e., floods, cyclones, droughts) are leading to the expansion of vector breeding grounds with increased transmission seasons [141]. These trends indicate a probable climate-driven spread of the malaria zone with serious implications for elimination in tropical regions and an increase in risk in the subtropical latitudinal zones [142].

### 8.4. Financing Gaps

Low global financing and chronic underfunding stand in the way of determining the scale-up of interventions [143]. WHO approximates that there is a shortfall in the annual funding of USD 3.1 billion against the USD 7.8 billion needed for the milestones towards achieving elimination by 2030 (WHO, 2024) [144]. Investment has been reduced by other competing global health crises, donor fatigue, and prioritization shifts in the geopolitical landscape [145]. Endemic countries have not raised enough money domestically and do not attract sufficient private sector participation. As a result, there is stasis in intervention coverage, ITN distribution, IRS campaigns, and case management activities, which are still sub-optimal. Similarly, resource constraints make research and development, particularly in the areas of new generation insecticides and vaccine development [146].

### 8.5. Unobserved Infections

The major impediment to elimination is persistence of low-density asymptomatic infections, most notably of *P. vivax* and submicroscopic *P. falciparum* reservoirs [147]. RDT is commonly insensitive in detecting these infections. Asymptomatic carriers are still capable of infecting mosquitoes, and they are not even aware of which chain of infections they belong to. Up to 30–40 percent of infections have not been recorded during surveillance in Southeast Asia and Latin America. To stop transmission, molecular diagnostics (PCR-based) and active population screening in the pre-elimination regions are necessary [148].

### 8.6. Urbanization and Migration

The malaria landscape is being changed by rapid urbanization, migration between countries, and population displacement. Poorly designed urban encroachment provides stagnant water bodies that are suitable for breeding by vectors [149]. New challenges in vector control logistics and follow-up of cases are also posed by migrant populations (often from endemic to non-endemic zones). The refugee crisis in sub-Saharan Africa, the Middle East, and South East Asia has brought malaria to poorly developed infrastructures [150]. Without cross-border surveillance and vector control activities in urban settings, malaria will be endemic in peri-urban areas [151].

## 9. Engaging and Shaping Future Directions and Policy Priorities

### 9.1. Integrated Vector Control Management (IVCM)

Relative freedom of reintroduced populations will require both chemical, biological, and environmental control strategies in the future. Considering the rapidity of resistance development, strategies using rotational application of insecticides, larval source control, and genetic vector control (e.g., sterile male or gene drive technologies) present a potential for overcoming resistance [152]. Integrated vector management also demands cross sectoral cooperation between the health, agriculture, and environment sectors [153].

### 9.2. Next-Generation Vaccines and Therapies

Novel drug candidates that seek to overcome resistance and to block transmission include cipargamin, ganaplacide, and compounds of the MMV [154]. In the development of pre-clinical studies of multivalent and messenger RNA-based vaccines against malaria, optimism continues to be well placed for improved efficacy and more lasting immunological protection compared to currently available vaccine tools. Equitable access to innovations will be a key component for successful continuity [155].

### 9.3. Combining Genomic and Digital Surveillance

With the help of digital platforms, combined with molecular diagnostics, transmission and resistance are mapped in real time [156]. Widespread genomic surveillance, long-term vector species dynamics, and parasite mutations may supply important data for focused design of evidence-based therapeutic interventions. These technologies are only useful if there is investment in local laboratory capacity [157].

### 9.4. Strengthening of Health Systems

Elimination efforts are based on resilient health systems that can provide universal access to diagnosis, treatment, and prevention. Integration of malaria services into primary healthcare, better supply chains, and community health workers programs may fill present gaps [158]. This buildup in laboratory networks and data reporting would increase accountability and better allocation of resources [159].

### 9.5. Adaptation to Climate

With the changing climate, adaptive surveillance approaches should be able to detect where new risk areas will emerge. Regional cooperation is critical for the prevention of cross-border transmission, especially in regions where there are a lot of mobile populations [160]. Future malaria control and prevention measures will therefore require the creation of climate-resilient infrastructure, the improvement of early warning systems, and the refinement of vector surveillance systems [161].

### 9.6. Equity and Community Engagement

Community involvement is still the key to effective malaria programs. Education, local ownership, and gender-sensitive interventions improve compliance and sustainability. Along with community volunteers, empowering women is also a key behavior change tactic that ensures that prevention tools like bed nets are properly used and retained [162].

### 9.7. Global Technical Strategy 2021–2030

The WHO Global Technical Strategy includes ambitious targets: achieve a reduction in malaria disease incidence, a 90% reduction in mortality, zero malaria in at least 35 countries, and elimination in all malaria-free countries by 2030. These goals can only be reached through an integrated approach that includes research, innovation, finance, and social justice. About interventions, the roadmap is characterized by universal access, country-specific adaptations, and continued political commitment [163].

### 9.8. Global and Regional Successes in Malaria Reduction

From 2019 to 2023, Rwanda has greatly expanded programs for prevention, diagnosis, and treatment of malaria with the support of partners including WHO and the Global Fund, PMI (U.S. President’s Malaria Initiative), and national programs, which helped reduce its malaria caseload by some 85% [26]. Liberia has also made great progress on reducing malaria cases (approximately 44% since 2017) through improved surveillance (strength and effectiveness) and case management. These are success stories of how global funding and technical support, accompanied by a long term national commitment, can see the burden of diseases drastically reduced even in the most high transmission settings [164].

In the WHO Southeast Asia Region, malaria cases have been reduced drastically, e.g., India has seen a 69% reduction in the estimated number of malaria cases between 2017 and 2023. In the Greater Mekong sub-region (Cambodia, Lao PDR, Myanmar, Thailand, Viet Nam), malaria incidence decreased by around 97% and mortality by over 99% between 2000 and 2020, after a cross-border collaboration, adjusting drug policy and implementing joint elimination strategies [165].

These decreases indicate a coordinated regional approach with support of WHO, national ministries of health, and donor partnerships, which are making us see that regional collaborative measures work across borders. Several countries in the Americas have eliminated malaria transmission and have been certified malaria free, including Paraguay (2018), Argentina (2019), El Salvador (2021), and Belize (2023) [166].

These achievements have been based on years of investments in surveillance, vector control, and strengthening of health systems, often in collaboration with national governments, fully aware and acting together with PAHO/WHO and international donors. Egypt was later certified by the WHO itself in the year 2024 as free from malaria; this is one of the few countries in the region to achieve such a huge goal, after struggling for years every time with zero indigenous cases. Egypt’s progress is a huge lesson in how it is possible to eradicate malaria in historically endemic regions with strong prevention programs in place, cross-sectoral cooperation, and informative surveillance.

## 10. Conclusions

Malaria is one of the oldest and most enduring plagues of humankind. Yet it remains one of the most preventable and curable diseases, whose impact on health and development continues to be staggeringly high among the poorest and most vulnerable communities. During the past 2 decades, a global-concerted effort has made significant progress, but the stagnation and new dangers showing are a reminder of the fragility of those advances. But no matter how great the scientific breakthroughs are, such as ACTs, RDTs, and vaccines, it is only through a robust health systems strategy, stable funding, and community involvement that the potential can be achieved. The next decade will be crucial in deciding whether malaria elimination goes back to fantasy or becomes a reality. To be successful, stakeholders will need to address the issue of resistance, invest in innovation, and adapt to the realities of climate and ensure that there is equity in access and resources. The goal of a malaria-free world requires global solidarity, long-term investments, and sustained commitment. By combining scientific advances with social and political intervention, humanity should be able to make malaria a preventable relic of the past, fulfilling one of the grandest public health success stories in the 21st century. Effective disease prevention and management strategies are worldwide and multifactorial. However, their successful use entails adaptation to local epidemiological realities, with health education incorporating local realities to facilitate primary, secondary, and tertiary prevention measures.

## Figures and Tables

**Table 1:** The regional trends of malaria according to the recent malaria WHO report 2023.

WHO Region	Estimated Cases (Million)	Incidence (per 1000 at Risk)	Malaria Mortality Rate (Deaths per 100,000 Population at Risk)	Malaria Deaths (Absolute Number)	Change in Malaria Cases/ Deaths (2000–2023)	Main Comments
African Region	246	227	52	569	Overall declined, plateaued since 2015 onwards	High-burden countries drive 94% of global cases; includes 46 countries such as Nigeria, Democratic Republic of Congo, Uganda, Ghana, etc. Cabo Verde was certified malaria-free in 2023.
South East Asia	4	2.3	0.3	6	87% cases since 2000	India and Indonesia are the main contributors; progress was also seen in Bangladesh, Thailand, and Myanmar.
Eastern Mediterranean	10.2	17.9	3.2	18	137% since 2015	Major surge in Pakistan (2022 floods, 10-fold rise in deaths) and Yemen; rising cases in Afghanistan, Sudan (~50% of regional deaths in 2023), Somalia, and Iran (local re-emergence). Other contributing factors: shortages of control commodities, funding gaps, and incomplete reporting due to political unrest.
Americas	0.55	3.6	0.2	0.34	65% cases since 2000	Belize was certified malaria-free in 2023; other countries include Brazil, Colombia, Venezuela, and Haiti.
Western Pacific	1.7	2.3	0.4	3.4	35% since 2015	PNG and Solomon Islands resurgence
Europe	0	0	0	0	Stable (Malaria-free)	Stable (malaria-free; no indigenous transmission).
Global Total	263	60.4	13.7	597	Plateau post 2015	Plateaued progress globally post-2015 with uneven regional trends.

**Table 2:** Data obtained from the WHO World Malaria Report 2024 (including data for 2023) [24] and from a complementary survey conducted by Hyun-II Shin et al., 2024 [25].

Metric	Global Value 2023	Notes	Source/Reference
Estimated malaria cases (Number of new + existing cases in endemic countries in 2023)	263 million	Total number of new + existing malaria cases in endemic countries in 2023	WHO World Malaria Report 2024 [24]
Incidence rate (Cases per 1000 population at risk in 2023)	60.4 cases	Annual number of cases per 1000 people at risk)	WHO World Malaria Report 2024 [24]
Number of Malarial Deaths	597,000	Absolute number of deaths due to malaria globally in 2023	WHO World Malaria Report 2024 [24]
Prevalence (children in high burden settings)	20 50%+ (among children under 5 in parts of sub-Saharan Africa)	Approximate proportion of children under 5 infected in parts of sub-Saharan Africa	World Malaria Report 2023 (Status of the World Malaria in 2022) [25]

**Table 3:** Contains information about the vector control interventions, their limitations, and recent advancements.

Interventions	Coverage Africa Households/Localized	Challenges/Limitations	Recent Advancement	Mode of Action
ITNs	More than 60% households	Insecticide resistance	PBO treated nets	Physical and chemical barrier
IRS	Localized	Costly	Clothianidin based long lasting sprays	Killing indoor resting mosquitoes
Larval site management	20% household	Environment dependent	biolarvicides	Targeting breeding sites

## Data Availability

The data generated in the work are presented in the manuscript.
